# Species-specific vulnerability of RanBP2 shaped the evolution of SIV as it transmitted in African apes

**DOI:** 10.1371/journal.ppat.1006906

**Published:** 2018-03-08

**Authors:** Nicholas R. Meyerson, Cody J. Warren, Daniel A. S. A. Vieira, Felipe Diaz-Griferro, Sara L. Sawyer

**Affiliations:** 1 BioFrontiers Institute, Department of Molecular, Cellular, and Developmental Biology, University of Colorado Boulder, Boulder, CO, United States of America; 2 Department of Microbiology and Immunology, Albert Einstein College of Medicine, Bronx, NY, United States of America; University of North Carolina at Chapel Hill, UNITED STATES

## Abstract

HIV-1 arose as the result of spillover of simian immunodeficiency viruses (SIVs) from great apes in Africa, namely from chimpanzees and gorillas. Chimpanzees and gorillas were, themselves, infected with SIV after virus spillover from African monkeys. During spillover events, SIV is thought to require adaptation to the new host species. The host barriers that drive viral adaptation have predominantly been attributed to restriction factors, rather than cofactors (host proteins exploited to promote viral replication). Here, we consider the role of one cofactor, RanBP2, in providing a barrier that drove viral genome evolution during SIV spillover events. RanBP2 (also known as Nup358) is a component of the nuclear pore complex known to facilitate nuclear entry of HIV-1. Our data suggest that transmission of SIV from monkeys to chimpanzees, and then from chimpanzees to gorillas, both coincided with changes in the viral capsid that allowed interaction with RanBP2 of the new host species. However, human RanBP2 subsequently provided no barrier to the zoonotic transmission of SIV from chimpanzees or gorillas, indicating that chimpanzee- and gorilla-adapted SIVs are pre-adapted to humans in this regard. Our observations are in agreement with RanBP2 driving virus evolution during cross-species transmissions of SIV, particularly in the transmissions to and between great ape species.

## Author summary

Multiple times, HIV-1 has entered the human population after emerging from a viral reservoir that exists in African primates. First, simian immunodeficiency virus (SIV) made the jump from monkeys into African great apes, and then from apes (namely, chimpanzees and gorillas) into humans. It is well appreciated that restriction factors, which are specialized proteins of the innate immune system, acted as host-specific barriers that drove virus adaptation during these spillover events. Here, we present data showing that a major constituent of the nuclear pore complex, RanBP2, was also a barrier to the spillover of SIVs, particularly in great ape species. Spillover of SIV into chimpanzee and gorilla populations required that the SIV capsid mutate to establish interaction with RanBP2 in the new host species. Our study highlights how essential housekeeping proteins, despite being generally more evolutionarily conserved than restriction factors, can also drive virus evolution during spillover events.

## Introduction

RanBP2 (also known as Nup358) is the major constituent of the cytoplasmic filaments extruding from the mammalian nuclear pore complex, where it mediates cargo import and export [1,2]. Depletion of RanBP2 negatively affects HIV-1 and HIV-2 infection and nuclear import [3–9]. Although HIV-1 can use other redundant pathways for import, pathways not involving RanBP2 lead to suboptimal chromosomal integration sites for the HIV-1 genome [4,7]. The interaction between RanBP2 and HIV-1 may not occur strictly at the nuclear pore. It was recently reported that the Kinesin-1 motor, KIF5B, relocalizes RanBP2 to the cytoplasm during infection [10]. As summarized in **Table 1**, RanBP2 seems to be generally important for the replication of HIV-1 and HIV-2, but not as important for the replication of simian immunodeficiency viruses (SIVs) from monkeys. RanBP2 depletion does not alter infection with SIVmac (macaques) [4,11], SIVmus (mustached monkeys) [8], SIVmon (mona monkeys) [8], or SIVcol (colobus guereza monkeys) [8]. One study reported mildly deleterious effects on SIVmac, SIVgsn (greater spot-nosed monkeys) and SIVmnd (mandrill) after RanBP2 depletion [8]. HIV-1 and HIV-2 are highly diverged from one another, and each share ancestry with separate lineages of monkey SIV, yet both require RanBP2 for optimal nuclear entry and genome integration [3,4,8,9,11–13]. This led us to hypothesize that evolution to bind and utilize RanBP2 is a necessary step in the successful transmission of SIVs to humans.

**Table 1:** Summary of published data on RanBP2 –capsid interactions^1^.

Virus capsid	Interacts with the Cyp domain of human RanBP2?	Infectivity diminished upon depletion of human RanBP2?	References
HIV-1	**Yes** 1. Size-exclusion chromatography 2. Isothermal titration calorimetry 3. Co-crystal structure solved 4. TRIM-RanCyp restriction assay 5. Co-sedimentation of purified components	**Yes** However, in mouse cells, deletion of only the RanCyp domain doesn’t affect HIV-1 [11]	[3–9,11–13]
HIV-2	**Yes** TRIM-RanCyp restriction assay	**Yes**	[8,9]
SIVmac macaque	**No** 1. TRIM-RanCyp restriction assay 2. Isothermal titration calorimetry	**No** [4,11] **Yes** [8]	[4,8,11]
SIVmon mona monkey	**No** TRIM-RanCyp restriction assay	**No**	[8]
SIVmus mustached monkey	**No** TRIM-RanCyp restriction assay	**No**	[8]
SIVgsn greater spot-nosed monkey	**No** TRIM-RanCyp restriction assay	**Yes**	[8]
SIVmnd mandrill	**No** TRIM-RanCyp restriction assay	**Yes**	[8]
SIVcol mantled guereza	**No** TRIM-RanCyp restriction assay	**No**	[8]
FIV cat	**Yes** 1. Isothermal titration calorimetry 2. TRIM-RanCyp restriction assay	**No**	[11,13]
MLV rodent	**No** TRIM-RanCyp restriction assay	**No**	[4,7,11]

^1^ Human lentiviruses are in rows shaded grey, primate lentiviruses in rows shaded blue, and non-primate lenti/retroviruses in rows shaded pink. The assays which have been used to substantiate each protein-protein interaction are also listed.

**Fig 1:**
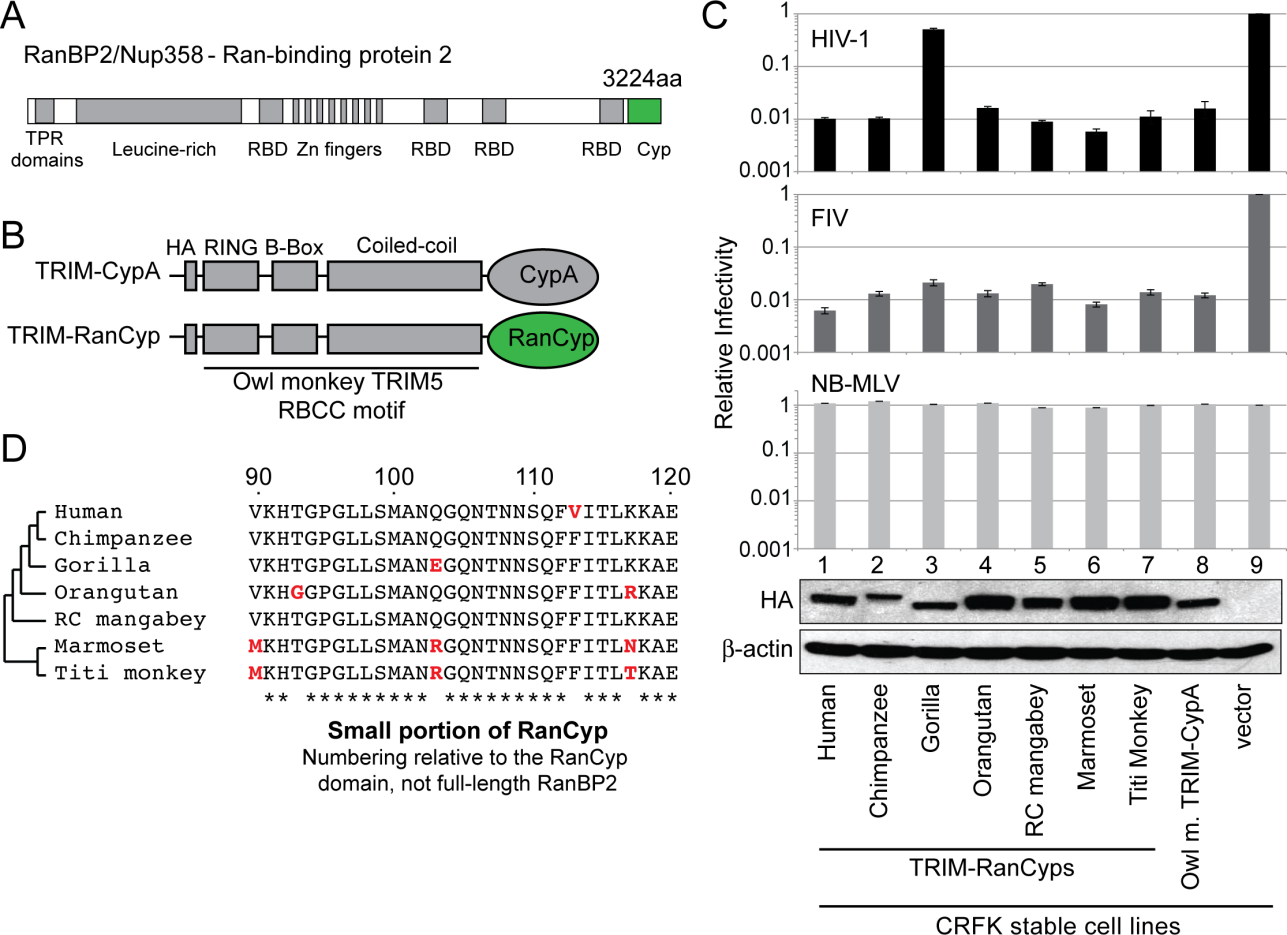
Not all primate RanBP2 proteins are the same. **A**) A domain diagram of RanBP2 is shown. TPR, tetratricopeptide; RBD, Ran binding domain; Cyp, Cyclophilin. **B**) Protein schematic of TRIM-fusion constructs showing the tripartite RING/B-Box/Coiled-coil (RBCC) domain fused to a cyclophilin domain. **C)** CRFK cell lines transduced to stably express different TRIM-RanCyps (bottom) were infected with VSV-G pseudotyped HIV-1, FIV, or NB-MLV, all encoding a GFP reporter. The percentage of cells infected in each sample was normalized to the empty vector control. A western blot detecting HA-tagged TRIM-RanCyp constructs is shown along with a β-actin loading control. Infections were performed in triplicate and error bars represent twice the standard error of the mean. **D)** Partial protein alignment of the RanCyp domain from primates in C. Here, residue coordinates refer to the coordinates of the human RanCyp domain (see exact numbering scheme in **S1 Fig**; 3060 needs to be added to these numbers to convert them to the residue position in full-length human RanBP2). Asterisks (*) indicate conserved residues. Red text indicates species-specific differences at non-conserved sites.

RanBP2 has a C-terminal cyclophilin domain that protrudes into the cytoplasm (herein, this domain is referred to as “RanCyp,” **Fig 1A**) [1,2,12]. The interaction with HIV-1 and HIV-2 is primarily mediated by this RanCyp domain [3,4,12], although other regions of RanBP2 may also be involved because RanBP2 lacking the RanCyp domain still retains residual interactions with capsid [3,11]. One study shows that, when expressed in mouse cells, human RanBP2 lacking the RanCyp domain can function equally to full-length RanBP2 in most measures of virus entry into the nucleus [11]. It’s possible that the RanCyp domain is not important in the context of infection in mouse cells. On the virus side, the cyclophilin-binding loop of capsid is primarily responsible for the interaction with RanBP2 [13]. Both the cyclophilin-binding loop (virus) and RanCyp (host) are highly variable in sequence [4,14,15].

One serious limitation in our understanding of RanBP2 as a host cofactor for viral replication is that all of the data summarized in **Table 1** are derived from the study of human RanBP2, even though many of these viruses are nonhuman viruses. For instance, theoretically it is possible that monkey SIVs are reliant on RanBP2 for nuclear import in monkey cells, but because they don’t interact with human RanBP2, no alteration in SIV infection is observed when RanBP2 is eliminated in human cells. In other words, SIVs may be adapted to the RanBP2 of their own species, but may have variable physical or genetic interactions with human RanBP2 that may or may not be biologically meaningful. This problem gets even worse when extrapolating the activities of human RanBP2 to non-primate retroviruses such as MLV of mice and FIV of cats. Here, we test the interaction of primate lentiviruses with the RanBP2 of their known host species.

In the current study we show that, indeed, not all primate RanBP2 orthologs are functionally equivalent. Following the theme of observations made with restriction factors, species-specific patterns of RanBP2 interaction exist, and these patterns depend on the specific virus that is being tested. We show that, in at least two instances involving African apes (chimpanzees and gorillas), the natural cross-species transmission of SIV to these species coincided with viral adaptation to interact with their RanCyp. Adaptation to bind RanCyp seems to have been an important evolutionary event that allowed SIV to jump into and then between great ape species, or that refined interactions with apes after these jumps occurred. While the importance of the RanCyp-capsid interaction has been debated, its restoration after two important cross-species transmission events lends credibility to its importance, particularly in the lineage of SIV that entered apes and ultimately humans to become HIV-1.

## Results

### Primate orthologs of RanBP2 differ in their interaction with HIV-1

We first employed a TRIM5 fusion assay to test for equivalence between different primate RanCyps. This assay has been used extensively to study the RanCyp-capsid interaction, and has been validated against many other types of biological assays (summarized in **Table 1**). The TRIM5 fusion assay exploits the architecture of the naturally-occurring owl monkey restriction factor TRIM-CypA [16,17]. In this assay, the cyclophilin domain (CypA) of this restriction factor is replaced with the cyclophilin domain of RanBP2 (RanCyp, **Fig 1A and 1B**). In cases where RanCyp interacts with capsid, the restriction activity of the TRIM portion of the molecule blocks virus replication, providing a quantitative readout of RanCyp–capsid interaction. First, we used retroviral transduction to generate CRFK (feline kidney) cell lines stably expressing the owl monkey TRIM-CypA restriction factor (positive control), or the hybrid TRIM-RanCyp (from human RanBP2, also a positive control), each fused to an HA tag. HIV-1 entry assays were then performed in these cell lines (see methods). Both TRIM-CypA and TRIM-RanCyp proteins drastically restricted HIV-1 infection (top graph, lanes 1 and 8 in **Fig 1C**) compared to a negative control cell line transduced with an empty vector (top graph, lane 9 in **Fig 1C**). Restriction of HIV-1 in this assay indicates that these cyclophilin domains are successfully interacting with the viral capsid. Additionally, both proteins equally restricted a second lentivirus, feline immunodeficiency virus (FIV), consistent with the observation that RanCyp is known to interact with the FIV capsid (middle graph, lanes 1 and 8 in **Fig 1C**) [11,13]. Neither restricted MLV, consistent with the observation that RanCyp does not interact with this virus (bottom graph, lanes 1 and 8 in **Fig 1C**) [4,7,11]. Collectively, these controls confirm previous findings that RanCyp mediates interaction with HIV-1 capsid, and that the TRIM-RanCyp assay detects this interaction.

We next tested the interaction between HIV-1 and the RanCyp of various nonhuman primate species. TRIM-RanCyps were constructed that represent the RanCyp domains of RanBP2 from chimpanzee (*Pan troglodytes troglodytes)*, gorilla (*Gorilla gorilla*), orangutan (*Pongo pygmaeus*), red-capped mangabey (*Cercocebus torquatus*), marmoset (*Callithrix jacchus*), and titi monkey (*Callicebus cupreus*) (**Fig 1D**; see **S1 Fig** for numbering scheme). Stable cell lines were constructed to express each of these HA-tagged chimeric proteins (**Fig 1C**, bottom), and then infected with HIV-1 (**Fig 1C**). Most primate TRIM-RanCyp proteins restricted HIV-1 by approximately 100-fold compared to the negative control, similar to the restriction by human TRIM-RanCyp. The one exception was gorilla TRIM-RanCyp, which restricted HIV-1 about 2-fold compared to the negative control (top graph lane 3, **Fig 1C**). Gorilla TRIM-RanCyp efficiently restricted feline immunodeficiency virus (FIV) suggesting that the hybrid protein is functional and that the loss of interaction with HIV-1 capsid is specific to HIV-1 (middle graph lane 3, **Fig 1C**). Thus, gorilla RanCyp does not interact with the HIV-1 capsid to the same extent as the other primate RanCyp domains tested.

**Fig 2:**
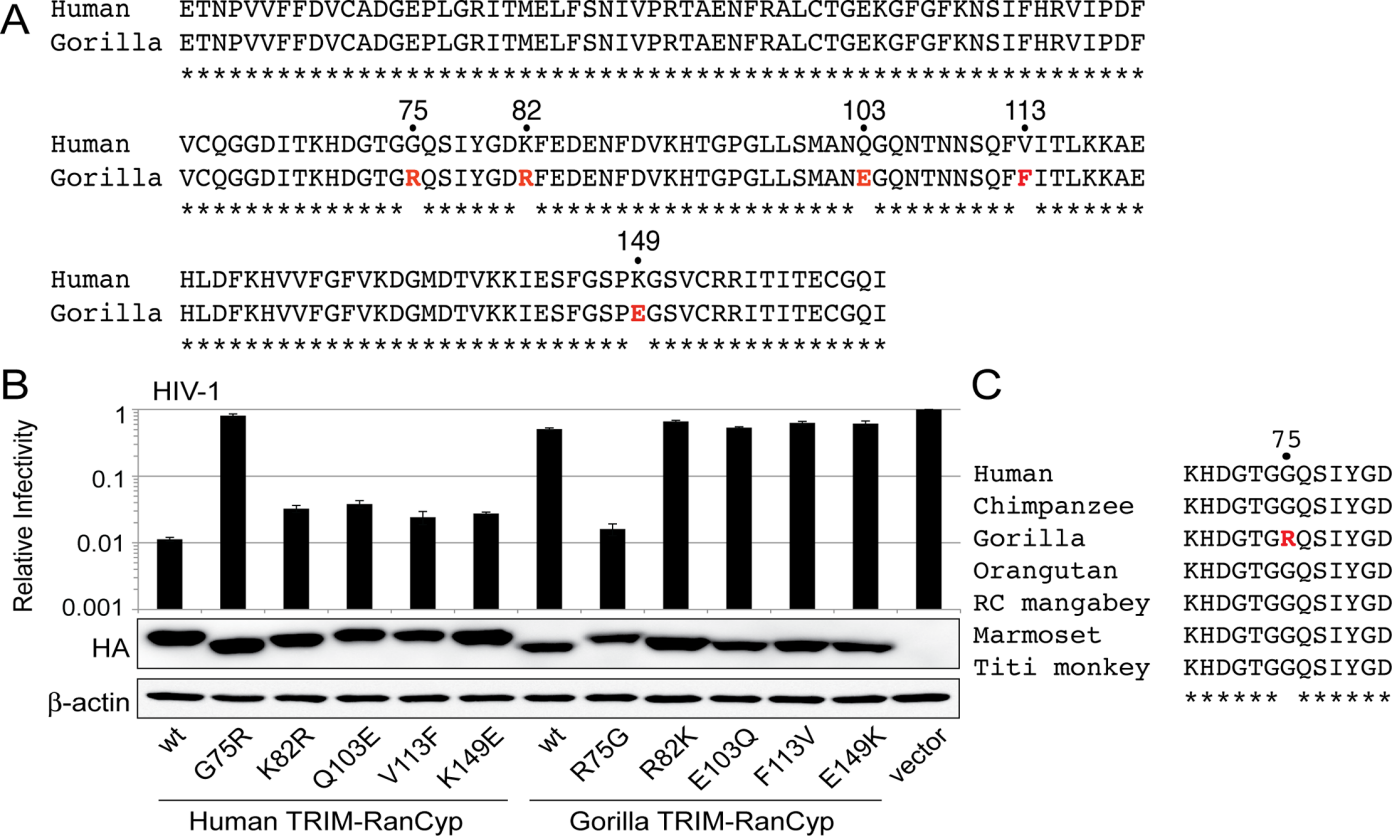
Evolutionary change at amino acid 75 in gorilla RanCyp alters interactions with HIV-1. **A)** Alignment of the RanCyp domain of human and gorilla RanBP2. Asterisks (*) indicate conserved amino acid positions. Red text indicates species-specific differences. **B)** CRFK cell lines stably expressing TRIM-RanCyp fusion proteins (bottom) were infected with VSV-G pseudotyped HIV-1 containing a GFP reporter. The percentage of cells infected in each sample was normalized to the empty vector control. A western blot detecting HA-tagged TRIM-RanCyp constructs is shown along with a β-actin loading control. Infections were performed in triplicate, and error bars represent twice the standard error of the mean. C) An alignment of the amino acids encoded at position 75 in the species included in this study. In all panels, the numerical amino acid coordinates refer to the RanCyp domain only. 3060 amino acids lie upstream of this domain in the full-length human RanBP2 protein.

Human and gorilla RanCyp are highly similar, differing by only five amino acids (**Fig 2A**). We made reciprocal substitutions of amino acids at these sites between human and gorilla RanCyp. Mutant TRIM-RanCyps were stably expressed in CRFK cells and then these cells were infected with HIV-1 (**Fig 2B**). We found that a mutation at site 75 plays a dominant role in reversing the phenotype of these two RanCyp domains. For instance, introduction of the gorilla residues 75R into the human RanCyp domain resulted in a 70-fold reduction in the ability to interact with HIV-1 capsid (**Fig 2B**). Reciprocally, introduction of the human RanCyp residue 75G increased the ability of gorilla RanCyp to interact with the HIV-1 capsid by 35-fold (**Fig 2B**). Mutations at the other four sites (82, 103, 113, and 149) that vary between human and gorilla RanCyp only resulted in a 1–3 fold change. Collectively, these data suggest that a single amino acid change at site 75 in the RanCyp domain can alter interactions with HIV-1 capsid. While some codons in this region of RanBP2 have experienced recurrent positive selection [4,14], residue 75 is not under positive selection and is conserved in all of the primates tested, except gorilla (**Fig 2C**).

### Gorilla-derived lentiviruses can interact with gorilla RanBP2

**Fig 3:**
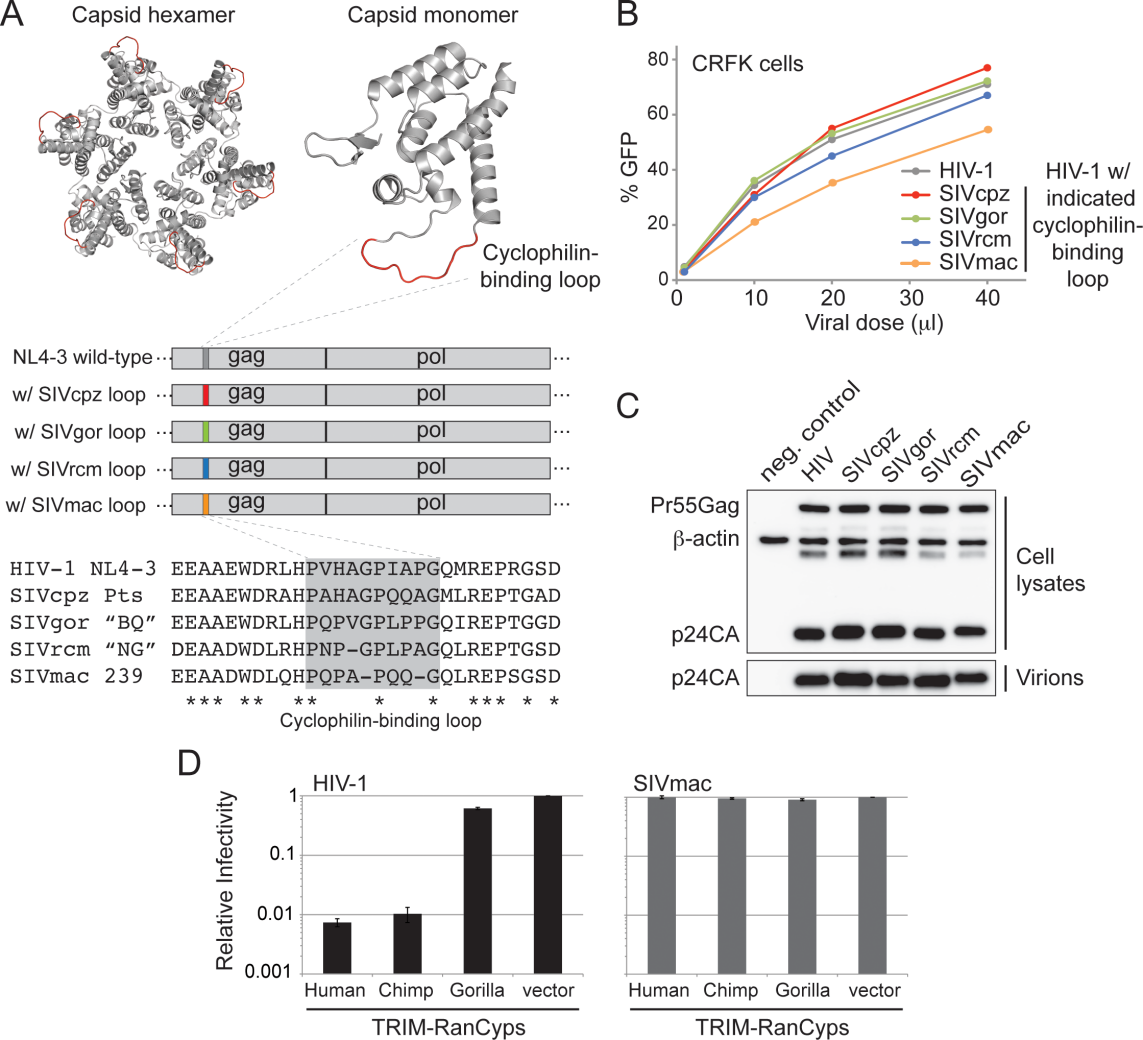
The construction of cyclophilin-binding loop substitutions in the HIV-1 capsid. **A)** (top) Crystal structures of hexameric (pdb:3GV2) and monomeric (pdb:1AK4) HIV-1 capsid. The region shown in red is the cyclophilin-binding loop. (middle) Location of the cyclophilin-binding loop within gag-pol. (bottom) An alignment of various cyclophilin-binding loops. Asterisks (*) indicate conserved residues. The cyclophilin-binding loop of HIV-1 group M was replaced with the corresponding loop (in gray box) from each indicated virus. **B)** VSV-G pseudotyped HIV-1 (containing cyclophilin-binding loop substitutions as indicated and a GFP reporter) was produced and used to infect CRFK cells. Percent infection is shown over a viral dose curve. Regression slopes were calculated for each curve and no significant differences were found for any pairwise comparison. **C)** Western blot of whole cell extracts and virions from virus-producing 293T cells. Viral packaging plasmids were transfected into 293T cells and samples were analyzed 48 hours post transfection by immunoblotting with anti-p24 and anti-β-actin. **D)** Single-cycle infection assays were performed in the indicated TRIM-RanCyp stable cell lines (bottom) with viruses that have the indicated cyclophilin-binding loop (top of graph) and a GFP reporter in the HIV-1 backbone. The percentage cells infected in each sample was normalized to the empty vector control. Infections were performed in triplicate and error bars represent twice the standard error of the mean.

Because HIV-1 does not interact with gorilla RanCyp, we wondered if this was also true for gorilla SIV (SIVgor). We generated mutants of HIV-1 group M where the 10 amino acid long cyclophilin-binding loop of capsid was replaced by the corresponding cyclophilin-binding loop from SIVgor (specifically, from the gorilla SIV isolate BQ664). We took this strategy in order to better isolate RanBP2-relevant differences between HIV-1 and SIVgor. An alignment of this cyclophilin-binding loop is shown in **Fig 3A**, where this specific SIVgor isolate is designated as SIVgor “BQ.” We also replaced the HIV-1 cyclophilin-binding loop with the corresponding cyclophilin-binding loop of SIVcpz (specifically from the chimpanzee subspecies *Pan troglodytes schweinfurthii;* denoted SIVcpzPts in **Fig 3A**) or SIVrcm (specifically from a Nigerian isolate of SIV isolated from red-capped mangabey; denoted SIVrcm “NG”). As a negative control, we replaced the cyclophilin-binding loop of HIV-1 with that found in SIVmac (representing macaque SIV isolate 239; denoted SIVmac 239). To ensure that HIV-1 containing each of these cyclophilin-binding loop substitutions was not compromised in function, we performed a series of controls. First, VSV-G pseudotyped virions of each hybrid virus were produced in 293T cells and titrated on CRFK cells. All of the yields were similar, with HIV-1 bearing the cyclophilin-binding loop of SIVmac being somewhat reduced compared to the HIV-1 control (**Fig 3B**). We also checked proper Gag (containing capsid) maturation in each cyclophilin-binding loop mutant virus by probing protein fractions isolated from 293T producer cells with an anti-p24 antibody. Equal expression and cleavage of Gag was observed for all virus mutants, both in cell lysates and in virions purified from cell supernatants (**Fig 3C**). SIVmac has previously been shown not to interact with human RanCyp (see **Table 1**). As expected, TRIM-RanCyp (from human RanBP2) did not interact with HIV-1 bearing the cyclophilin-binding loop of SIVmac (denoted “SIVmac” on the figure) (**Fig 3D**). This result, where the SIVmac cyclophilin-binding loop is isolated within the context of HIV-1, supports the conclusion that the cyclophilin-binding loop of HIV-1, but not SIVmac, engages RanBP2. It also supports the conclusion that the cyclophilin-binding loop of HIV-1 is required for interaction with RanBP2. We conclude that the assay used here can recapitulate known interactions between RanCyp and lentiviral capsids.

**Fig 4:**
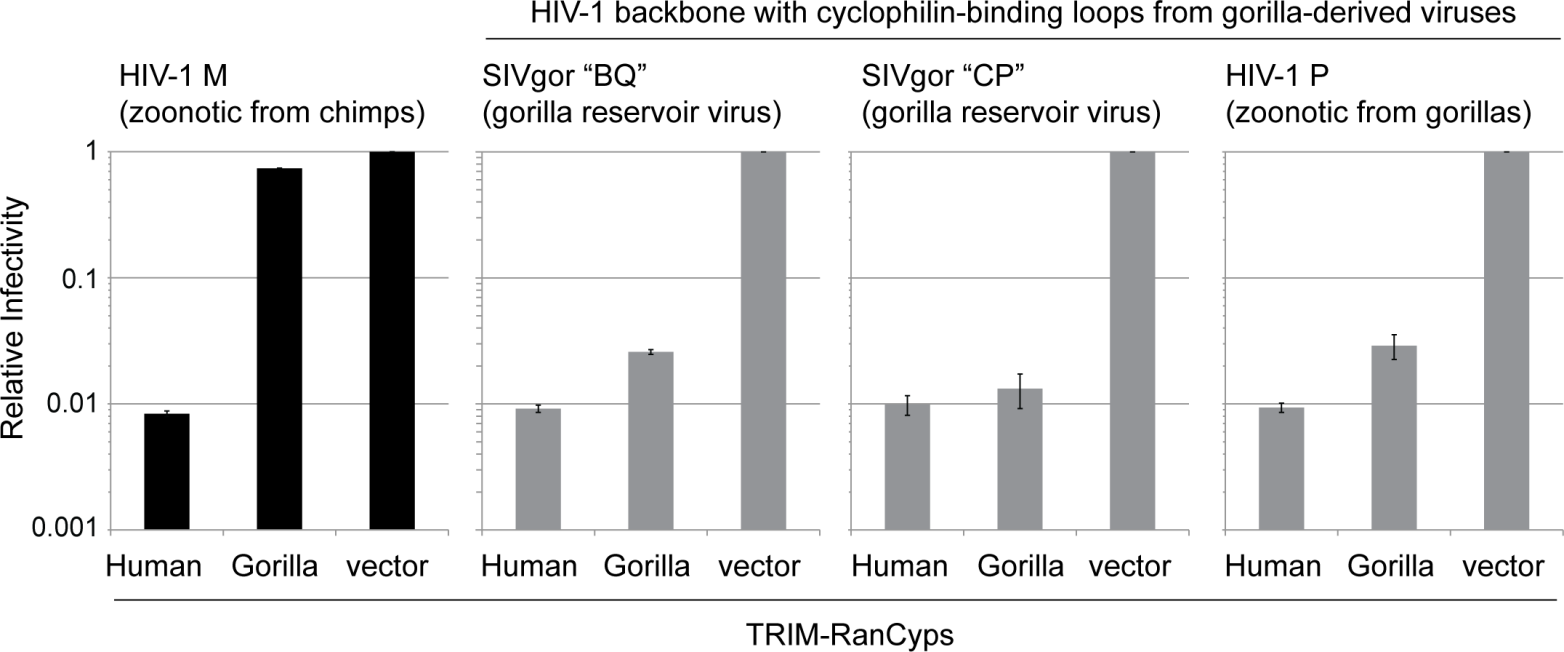
Cyclophilin-binding loops from gorilla-derived viruses interact with gorilla RanCyp. Cell lines stably expressing TRIM-RanCyp (as indicated across the bottom) were infected with HIV-1 bearing the indicated cyclophilin-binding loop (top) and a GFP reporter. The percentage of cells infected in each sample was normalized to the empty vector control. Infections were performed in triplicate and error bars represent twice the standard error of the mean.

This proof of principle allowed us to ask whether or not SIVgor interacts with gorilla RanBP2, given that HIV-1 does not. Using the system described above, we first tested the ability of the cyclophilin-binding loop of SIVgor to interact with gorilla RanCyp. We found that the cyclophilin-binding loop of SIVgor isolate BQ664 does interact with gorilla RanCyp, whereas the cyclophilin-binding loop of an HIV-1 group M virus does not (**Fig 4**). To confirm this result, we engineered HIV-1 to encode the cyclophilin-binding loop of another SIVgor isolate, CP684, and found that this virus could also interact with gorilla RanCyp. These SIVgor variants (SIVgorBQ and SIVgorCP) represent the clade of gorilla viruses from which HIV-1 groups P and O arose after zoonotic transmission from gorillas to humans [18]. (**S2 Fig** shows an alignment of all cyclophilin-binding loops used in this study.) Finally, we also engineered HIV-1 to encode the cyclophilin-binding loop from HIV-1 group P. The logic in this experiment is that SIVgor isolates BQ and CP, as well as HIV-1 group P, can all be thought of as gorilla-derived viruses. We find that the cyclophilin-binding loop of HIV-1 group P can also interact with gorilla RanCyp (**Fig 4**; right). This suggested to us that gorilla-derived viruses may have adapted to interact with the RanCyp domain of gorilla RanBP2. Further, this was an initial hint that there is a highly dynamic relationship between host RanBP2 and lentiviruses in African apes.

### Species-specific differences in RanCyp drove viral evolution during known cross-species transmission events

**Fig 5:**
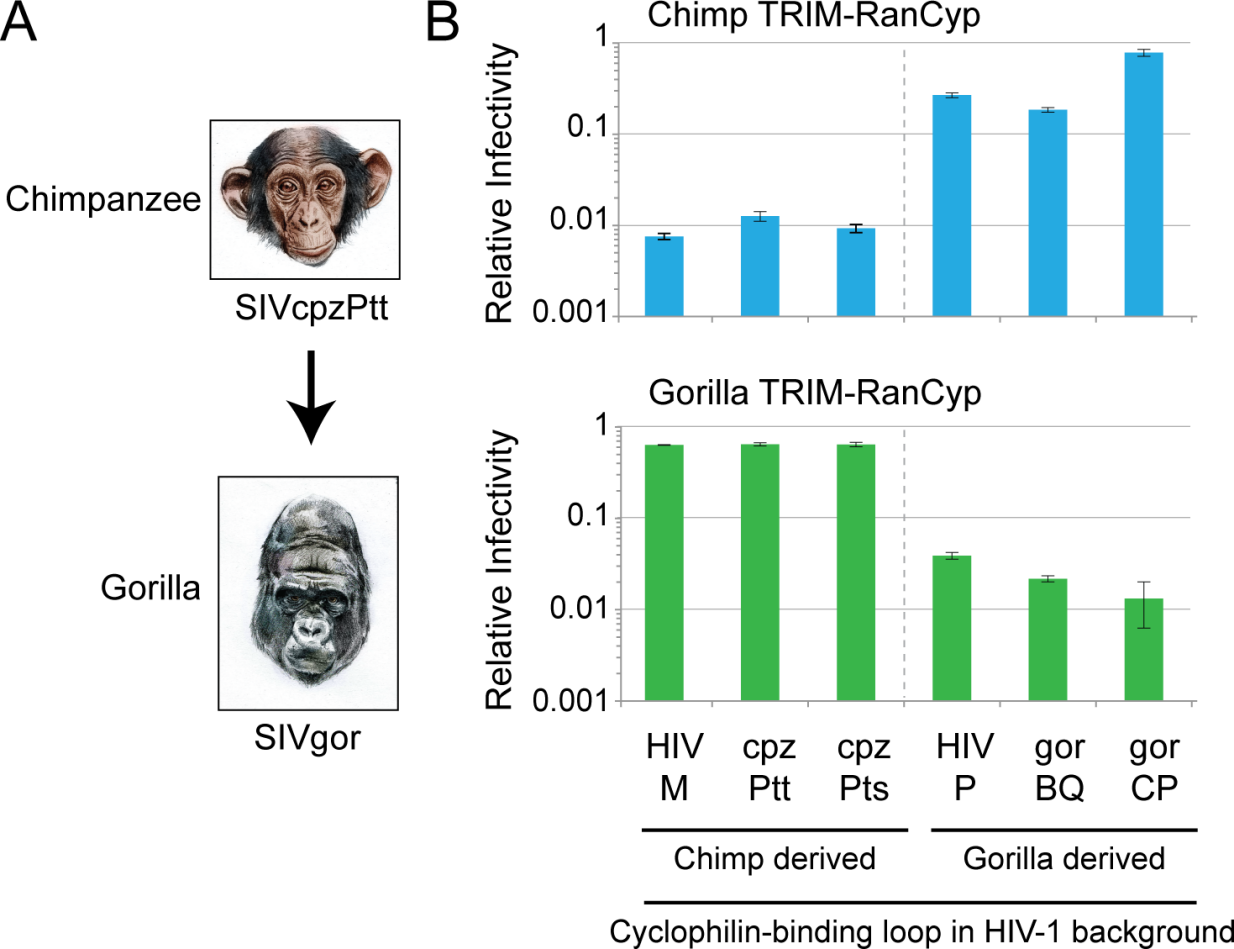
SIVcpz adapted to gorilla RanCyp during transmission from chimpanzees to gorillas. SIVgor was created by a cross-species transmission of SIVcpz from chimpanzees to gorillas [19,20]. CRFK cell lines stably expressing chimpanzee or gorilla TRIM-RanCyp (as indicated at the top of each graph) were infected with viruses encoding the indicated cyclophilin-binding loop (X-axis) in the HIV-1 backbone, and a GFP reporter. The percentage of cells infected in each sample was normalized to an empty vector control. Infections were performed in triplicate and error bars represent twice the standard error of the mean.

We next asked whether the ability of gorilla-derived SIVs to interact with gorilla RanBP2 was a trait that was gained as SIV transmitted into gorillas. Gorillas acquired SIV from chimpanzees. Specifically, SIVcpz was transmitted to gorilla populations from the *Pan troglodytes troglodytes* subspecies of chimpanzees (this version of SIVcpz is called SIVcpzPtt; **Fig 5A**) [19,20]. We next performed an experiment to determine if SIVcpz could already interact with gorilla RanBP2 before this transmission, or whether the virus had to adapt to do so during the spillover event from chimpanzees to gorillas. We cloned into HIV-1 group M the cyclophilin-binding loops of two chimpanzee-derived strains (SIVcpzPtt, SIVcpzPts) (see **S2 Fig** for alignments). Further, HIV-1 group M is derived from chimpanzees, and therefore can be thought of as another chimpanzee-derived virus. The cyclophilin-binding loops of all three chimpanzee-derived viruses are unable to interact with the RanCyp of gorilla RanBP2 (**Fig 5B**, columns 1–3). Further, the cyclophilin-binding loop of the three gorilla-derived viruses gained the ability to interact with gorilla RanCyp, but in the process lost interaction with chimpanzee RanCyp (**Fig 5B**, columns 4–6). Therefore, it appears that this cross-species transmission was accompanied by changes to capsid that resulted in RanCyp binding in the new host species, and loss of RanCyp interaction in the old host species.

**Fig 6:**
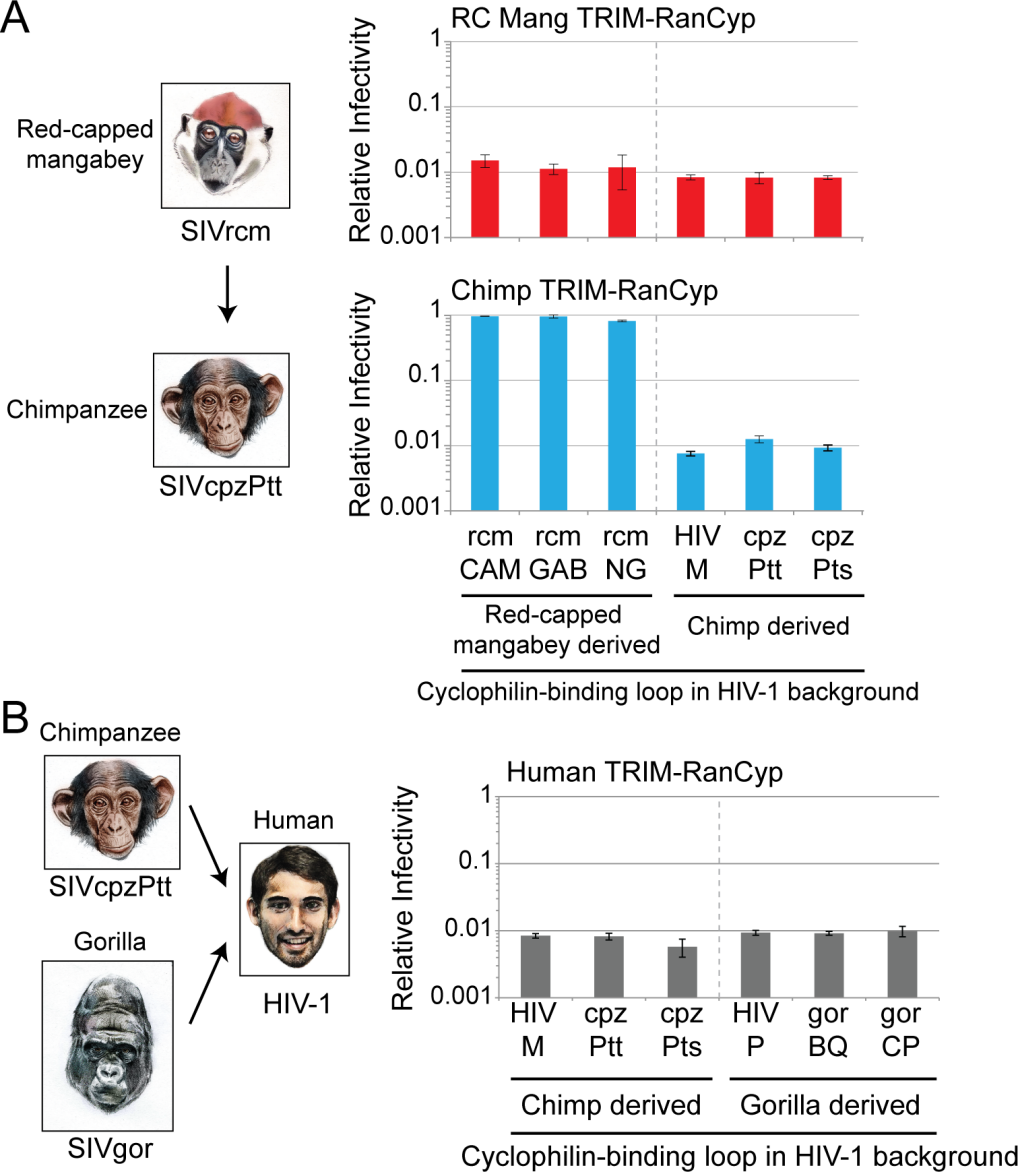
SIV adapted to bind RanCyp during its transmission from monkeys to apes, but not from apes to humans. **A)** The *gag* portion from SIVcpz was derived from SIVrcm after a cross-species transmission of a recombinant SIV into chimpanzees [21]. (But see [22] for extended discussion of this transmission event.) **B)** HIV-1 arose after several different zoonoses of SIV from chimpanzees and gorillas [18,23]. **A, B)** CRFK cell lines transduced to stably express the indicated TRIM-RanCyp (as indicated at the top of each graph) were infected with viruses encoding the indicated cyclophilin-binding loop (X-axis) in the HIV-1 backbone, and a GFP reporter. The percentage of cells infected in each sample was normalized to an empty vector control. Infections were performed in triplicate and error bars represent twice the standard error of the mean.

Backing up one step further, SIVcpz emerged as the result of recombination between SIV strains from multiple African monkey species [21,22]. The *gag* coding sequence (encoding capsid) of SIVcpz was thought to have been contributed by SIVrcm from red-capped mangabeys (**Fig 6A**) [21]. To determine if viral adaptation to chimpanzee RanBP2 might have occurred during this spillover event, we next cloned the cyclophilin-binding loops of three red capped mangabey-derived isolates (SIVrcmCAM, SIVrcmGAB, SIVrcmNG; see **S2 Fig** for alignments) into the HIV-1 reporter virus. The cyclophilin-binding loop of all three mangabey SIVs tested were able to interact with the RanCyp of mangabey but not chimpanzee (**Fig 6A**, columns 1–3). However, the cyclophilin-binding loop of all three chimpanzee-derived SIVs assayed had gained the ability to interact with chimpanzee RanCyp, and also retained interaction with mangabey RanCyp (**Fig 6A**, columns 4–6). This suggests that the SIV capsid evolved the ability to bind chimpanzee RanBP2 during the transmission from African monkeys to chimpanzees, a key transmission event that first introduced SIV into apes. Recently, the SIVrcm origins of SIVcpz *gag* has been called into question [22]. If and when other sources for this portion of SIVcpz are proposed, viral capsids and RanCyp from those species can be tested as well.

Finally, we wanted to test if viral adaptation to RanBP2 might have occurred during the spillover events that led to the emergence of HIV-1 in human populations. HIV-1 emerged in human populations following multiple independent zoonotic transmissions of SIVcpzPtt from chimpanzees and SIVgor from gorillas (**Fig 6B**) [18,23]. We find that the cyclophilin-binding loops of all chimp- and gorilla-derived viruses can interact with human RanCyp (**Fig 6B**, columns 1–6). Therefore, adaptation to utilize human RanBP2 was probably not a barrier to the zoonotic events that led to the emergence of HIV-1. In this way, it appears that chimp and gorilla RanBP2 could have provided an evolutionary stepping stone to infecting humans.

### Chimpanzee and gorilla SIVs use a RanBP2-dependent nuclear import pathway

**Fig 7:**
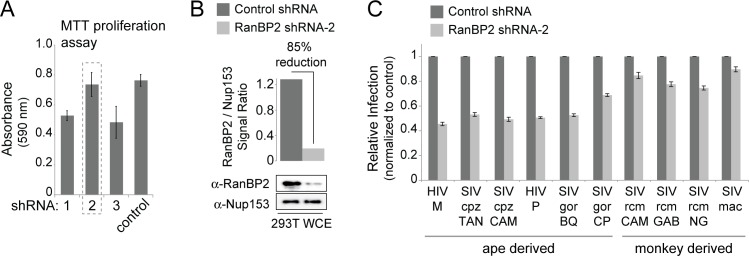
HIV-1, SIVcpz, and SIVgor are equally dependent on RanBP2. **A)** 293T cells were infected with lentivirus harboring the indicated shRNA constructs and MTT-proliferation assays were carried out in triplicate at 96 hours post infection. Absorbance was measured at a wavelength of 590 nm. Reduced absorbance is indicative of reduced cell proliferation. **B)** Whole cell extract (WCE) from 293T cells infected with lentivirus harboring the indicated shRNA construct was collected at 72 hours post infection and immunoblotted with antibodies raised against RanBP2 or Nup153 as a loading control. **C)** 293T cells treated with the indicated shRNA constructs were infected with HIV-1 bearing the indicated cyclophilin-binding loop (X-axis) and a GFP reporter. The percentage cells infected in each sample was normalized to the shRNA control. Infections were performed in triplicate and error bars represent twice the standard error of the mean.

As summarized in **Table 1**, RanBP2 seems to be generally important for nuclear import of HIV-1 and HIV-2, but so far there is less evidence that it is important for SIVs from monkeys. We next tested whether great ape SIVs are dependent on RanBP2 for optimal entry. We used a lentiviral shRNA system to knockdown RanBP2 in 293T cells (**Fig 7A and 7B**). The knockdown of RanBP2 is known to be toxic to cells [4,11], so we tested three different RanBP2 shRNA constructs for their general effect on cell proliferation using an MTT assay (see methods; **Fig 7A**). Compared to a non-targeting shRNA control, the RanBP2-targeting shRNA-2 construct had minimal toxicity (**Fig 7A**) and conferred approximately 85% knockdown of RanBP2 (**Fig 7B**). 293T cells transduced with either the non-targeting shRNA control or RanBP2-targeting shRNA-2 were then infected with our panel of VSV-G pseudotyped HIV-1 bearing various cyclophilin-binding loops. Cell populations were first gated for live cells, and then the percent infection was calculated by scoring for GFP-positive cells (**Fig 7C**). RanBP2 depletion caused an approximate 50% reduction in infection for most of the ape-derived viruses (HIV-1, SIVcpz, SIVgor). As expected, we did not observe a substantial effect of human RanBP2 knockdown on the monkey-derived viruses (all three SIVrcm strains and SIVmac), probably because these viruses do not interact with human RanBP2. Thus, it seems that ape-derived SIVs are dependent on RanBP2 for nuclear entry, to the same degree that HIV-1 is dependent on RanBP2. This raises the speculation that evolution to utilize RanBP2-dependent entry pathways was a novel adaptation acquired as SIVs entered apes from the monkey reservoir. To definitively prove this, knockdown of monkey RanBP2 would need to be conducted in monkey cells, and then the impact on monkey-derived SIV infection assessed.

## Discussion

Our data suggest that gained physical interactions between SIV capsids and the RanCyp domain of RanBP2 correlate with known spillover events into and between apes. Because RanBP2 varies from one primate species to the next, SIVrcm and SIVcpz had to adapt to bind the RanBP2 of chimpanzees and gorillas, respectively. Our results are consistent with a scenario where some cross-species transmissions of primate lentiviruses, particularly to and between African apes, required the adaptation of capsid to interact with the RanCyp domain of RanBP2 encoded in the new host species. One could argue that the work of Meehan and colleagues [11], performed with human RanBP2 in mouse cells, suggests that the RanCyp domain may not be important to HIV-1 at all. This conclusion is called into question by the otherwise unlikely observation that every chimpanzee and gorilla virus we have studied demonstrates a gain-of-interaction with the RanCyp domain of its new host species. Our findings do not rule out the possibility that other regions of RanBP2 or capsid are involved in their mutual interaction. Indeed, two important studies have shown that interactions between capsid and RanBP2 are maintained even in the absence of the RanCyp domain [3,11]. One possibility is that the interaction that we have isolated and studied in this work provides a level of specificity to an interaction that actually involves a more elaborate interaction surface.

The cyclophilin-binding loop and surrounding regions of capsid interact with several proteins in the host cell, including MxB, CPSF6, TRIM5α, and cyclophilin A (CypA) [24–28]. During a spillover event into a new host species, any of these host interactions (or all of them) that don’t function correctly in a new host would be predicted to drive virus evolution. At the most basic level, our study has shown that RanBP2 is not functionally equivalent between species, and therefore has the potential to act as a selective force driving evolution of the SIV capsid. This is also likely to be true for TRIM5α, which is highly host species-specific in its interactions with lentiviruses [29,30]. However, MxB, CPSF6, and CypA are not likely to drive capsid evolution as lentiviruses move from one primate species to the next. This is because these three host proteins are predicted to function identically between primate species with respect to lentiviral interactions, for the following reasons. First, the capsid binding domain in CPSF6 is perfectly conserved between primate species [26]. Second, the CypA protein sequence has remained unchanged during ~30 million years of primate evolution (**S3 Fig**). Third, MxB is under positive selection, but there is no overlap between these variable residue sites and those mapped to dictate lentiviral restriction [31]. Therefore, interactions between lentiviruses and the CPSF6, MxB, and CypA proteins are predicted to be equal in all primate species. It seems reasonable to conclude that the evolution of the cyclophilin-binding loop in capsid during spillover is primarily driven by TRIM5α and RanBP2, but not the other factors known to interact with this loop.

The transmission of SIV from monkeys to apes was a key event that set the stage for zoonosis to humans. In the case of RanBP2, we show that SIV capsid evolved to interact with the RanCyp domain of RanBP2 upon transmission to both chimps and gorillas, but once SIVgor and SIVcpz had become established, these viruses subsequently did not need to evolve further to utilize human RanBP2. This pattern of viral adaptation to RanBP2 directly mirrors what has also been reported about viral adaptation to APOBEC3G. Similarly, APOBEC3G was previously shown to have acted as a barrier in the monkey-to-chimpanzee, and chimpanzee-to-gorilla, transmissions of SIV, but not subsequently in the transmission of chimpanzee viruses to humans [18,32]. In other ways, the RanBP2 and APOBEC3G findings differ. Because the usage of the RanBP2-mediated import pathway may not be as important for the biology of SIV in African monkeys, it is interesting to speculate that this entire viral import pathway was a novel adaptation of SIV as it entered ape species. However, this speculation is not yet well supported and a more in-depth study of interactions between monkey SIVs and their host RanBP2 molecules in monkey cells will be necessary in order to gain additional insight into the nuclear import of these viruses. APOBEC3G restriction, on the other hand, is relevant to all HIV and SIVs studied.

Our current study is the first to provide functional evidence that divergence in post-entry cofactors for viral replication, provided by the host, can drive changes in SIV/HIV genomes during spillover. The nuclear pore and the cellular process of nuclear trafficking are both common targets of antagonism and utilization by viruses [33]. One can imagine intense selective pressure at play to keep viruses out of cells altogether via receptor evolution [34–40] or, when that fails, out of the nucleus via evolution of the nuclear transport machinery. It’s possible that nuclear pore components, like cellular entry receptors, are another class of host cofactor proteins that often create critical barriers to viral host switching, thus driving viral evolution in the process. RanBP2 adds to a growing list of dynamic cellular host proteins that exert selective pressure during the spillover of viruses in nature [34–38,41–48].

## Materials and methods

### Cell lines

HEK293T cells (ATCC #CRL-3216) and CRFK cells (feline kidney, ATCC #CCL-94) were cultured in Dulbecco’s modified Eagle media (Sigma, #D6429) supplemented with 10% fetal bovine serum (Sigma, #F2442), 2 mM L-glutamine (Invitrogen, #25030–081), and 1% antibiotics (Corning, #30–002). Cells were cultured at 37°C and in 5% CO_2_. Primary and immortalized cell lines from primate species (see **S4 Fig**) were grown in Dulbecco’s modified Eagle media (fibroblasts) or RPMI-1640 (B-Lymphocytes; Sigma, #R0883) supplemented with 15% fetal bovine serum, 2 mM L-glutamine, and 1% antibiotics. Cells were cultured at 37°C and in 5% CO_2_.

### Primate *RanCyp* and *CypA* gene sequences

The method of acquisition for all primate gene sequences is summarized in **S4 Fig**. Human RefSeq sequence was obtained from the NCBI nucleotide database. Chimpanzee, Sumatran orangutan, and rhesus macaque gene sequences were obtained from the UCSC genome database (http://genome.ucsc.edu/) using the BLAT tool. Additional *RanCyp* and *CypA* sequences were sequenced from cDNA generated using Superscript III First-Strand Synthesis System (Thermo, #18080051) with oligo(dT) primers. PCR was performed with Phusion High Fidelity PCR Master Mix (NEB, #F-531S). Details of the PCR and sequencing strategy, along with primer sequences, are given in **S1 Table**. Primate gene sequences generated in this study have been deposited in GenBank and are listed in **S4 Fig**.

### TRIM-CypA and TRIM-RanCyp expression constructs

HA-tagged owl monkey TRIM-CypA in the pLPCX expression vector was a kind gift from Michael Emerman. This TRIM-CypA construct is representative of the naturally occurring fusion protein from *Aotus trivirgatus* (GenBank accession #AAT73777) [16,17]. TRIM-RanCyps were constructed by generating 20–25 base pair overlapping regions of owl monkey TRIM-CypA and RanCyp. Nucleic acids from primate species shown in **Fig 1** were used as templates in PCR reactions to generate RanCyp. Details of the primate cell lines and PCR strategy and be found in **S4 Fig** and **S1 Table**, respectively. Overlapping fragments were spliced together in a PCR reaction using each fragment as a template and outside flanking primers. Constructs were TA-cloned into pCR4 (Invitrogen, #K4575-01). An N-terminal HA tag was added in a PCR reaction and these tagged constructs were TA-cloned into the gateway entry plasmid pCR8 (Invitrogen, #K2500-20). An LR Clonase II reaction (Invitrogen, #11791–100) was used to move these constructs into a Gateway-converted pLPCX retroviral vector (Clontech, # 631511) via a recombination reaction. TRIM-RanCyp mutants were generated using PfuTurbo DNA polymerase (Stratagene, #600250). Parental pLPCX plasmids were used as a template along with primers (**S1 Table**) containing the mutations of interest.

### Generation of stable cell lines

In order to make cell lines that stably express owl monkey TRIM-CypA and TRIM-RanCyps, retroviral vectors were used to transduce CRFK cells. To generate the retroviral vectors, 293T cells were seeded at a concentration of 1x10^6^ cells/well in a 6-well dish. 24 hours later each well was transfected with 2 μg pLPCX construct (empty or encoding the gene fragment of interest), 1 μg pCS2-mGP encoding MLV gag-pol [49], and 0.2 μg pC-VSV-G at a final 1:3 ratio of DNA to TransIT-293 (μg DNA: μl TransIT-293). Supernatants were collected after 48 hours, passed through a 0.2 μm filter, and used to infect CRFK cells. After 24 hours, media containing 8 μg/ml puromycin was added to select for transduced cells. Cell lines were expanded and grown in puromycin for at least two weeks before expression of TRIM-CypA and TRIM-RanCyp constructs was detected by western blot.

### Antibodies and western blot analysis

Stable CRFK cell lines or 293T cells transduced with shRNA lentivirus were grown to confluency in a 6-well dish, collected using a cell scraper, and lysed in either a buffer containing 150 mM NaCl, 50 mM Tris-HCl (pH 7.4), 1% NP-40, and Complete protease inhibitor (Roche, #11836170001)(stable CRFK cell lines) or RIPA buffer supplemented with Complete protease inhibitor (293T cells). CRFK cells were rotated in lysis buffer at 4°C for 45 minutes and whole cell extracts were cleared in a microcentrifuge by spinning at maximum speed for 15 minutes. 293T cells were sonicated on ice in RIPA buffer using the Qsonica Q500 (sonication settings: microtip, 40% amplitude, 7 second pulse). After quantitation of protein concentration using a Bradford assay, 20 μg of whole cell extract was resolved using a 10% (HA-TRIM-RanCyp and Gag) or 7.5% (endogenous RanBP2) polyacrylamide gel and transferred to a nitrocellulose membrane. To detect endogenous RanBP2, transfer was done at 400 mA for 2 hours in buffer containing 0.02% sodium dodecyl sulfate. HA-tagged constructs were detected using a 1:5000 dilution of mouse anti-HA antibody conjugated to horseradish peroxidase (Roche, #12013819001). Maturation of Gag protein was monitored using a 1:1000 dilution of mouse anti-p24 (AIDS reagent database, #183-H12-5C). Endogenous RanBP2 was detected using a 1:1000 dilution of rabbit anti-RanBP2 (Thermo Fisher Scientific, #PA1-082). Endogenous β-actin was detected as a loading control using a 1:1000 dilution of mouse anti-β-actin (Santa Cruz, #sc-47778). Endogenous Nup153 was detected as a loading control using a 1:1000 dilution of mouse anti-Nup153 (Covance, #MMS-102P). A 1:10,000 dilution of goat anti-mouse horseradish peroxidase-conjugated antibody (Thermo Fisher Scientific, #32430) or goat anti-rabbit horseradish peroxidase-conjugated antibody (Thermo Fisher Scientific, #32460) was used as a secondary probe. Blots were developed using the ECL Plus detection reagent (GE Healthcare, #RPN2132).

### Single-cycle infection assays

Viruses for single-cycle infection assays were packaged in 293T cells by co-transfection of plasmids encoding viral proteins and VSV-G, along with a transfer vector, as follows: HIV-1 and cyclophilin-binding loop mutants (pMDLg/pRRE, pRSV-Rev, pMD2.G, pRRLSIN.cPPT.PGK-GFP.WPRE; all available on Addgene), FIV (pFP93 [50], pC-VSV-G, pGIN-SIN:GFP [50]), NB-MLV (pCS2-mGP [49], pC-VSV-G, pLXCG). After 48 hours, supernatant containing viruses was harvested, filtered, and frozen. Viruses were titered on CRFK cells by measuring percent GFP-positive cells along a volume gradient of virus supernatant. For infection assays, CRFK stable cells lines were plated at a concentration of 7.5x10^4^ cells/well in a 24-well plate and infected with HIV-1, cyclophilin-binding loop mutants, FIV, or NB-MLV such that 30–50% of the control cell line was infected. Two days post-infection, cells were fixed in 2% paraformaldehyde for 15 minutes, washed three times with 2 mL FACS buffer (DPBS supplemented with 2% FBS and 1 mM EDTA), resuspended in 500 μl FACS buffer, and analyzed by flow cytometry for expression of GFP using the BD Bioscience Fortessa cell analyzer. All infections were performed in triplicate using a single virus stock, and all results were confirmed using at least two experimental replicates.

### shRNA lentivirus production and RanBP2 knockdown

Lentivirus harboring Sigma-Aldrich MISSION shRNA constructs (#TRCN0000272800, labeled ‘shRNA-1’ in **Fig 7**; #TRCN0000272801, labeled ‘shRNA-2’ in **Fig 7**; #TRCN0000003453; labeled ‘shRNA-3’ in **Fig 7**; #SHC002, labeled ‘control’ in **Fig 7**; all shRNA constructs were obtained from the Functional Genomics facility at University of Colorado Denver) were packaged in 293T cells by co-transfection of pLKO.1 (shRNA packaging plasmid), pMDLg/pRRE, pRSV-Rev, and pMD2.G (the latter three plasmids are described above). After 48 hours, supernatant containing viruses was harvested, filtered, and frozen. Viruses were titered on 293T cells by measuring RanBP2 knockdown efficiency via western blot 72 hours post infection. Images were quantified using ImageJ and samples were normalized using a Nup153 loading control (**Fig 7B**). For infection assays, 293T cells were plated at a concentration of 3.0x10^5^ cells/well in a 12-well plate and infected with shRNA lentivirus. Two days post-infection, cells were re-seeded at a concentration of 7.5x10^4^ cells/well in a 48-well dish. After an overnight incubation single-cycle infection assays using VSV-G pseudotyped HIV-1 and cyclophilin-binding loops mutants were carried out as described above. Simultaneously, a proliferation assay using MTT (Thermo Fisher, #M6494) was performed to determine relative toxicity of shRNA constructs. Cells were incubated with MTT solution (1:1 mixture of serum-free media and 5 mg/mL MTT in PBS) at 37°C for 3 hours and reactions were then solubilized with MTT solvent (4 mM HCl and 0.1% NP-40 in isopropanol) by shaking at room temperature for 15 minutes. Absorbance at 590 nm was measured for each sample.

### Cyclophilin-binding loop capsid mutants

pMDLg/pRRE expressing HIV-1 gag-pol was used as a template for site-directed mutagenesis using PfuTurbo DNA polymerase (Stratagene, #600250). Cyclophilin-binding loop sequences can be found in **S2 Fig** and primers used in site-directed mutagenesis PCR are listed in **S1 Table**. Purification of virions to probe Gag maturation of capsid mutants was performed by layering 500 μl of filtered supernatant from virus-producing 293T cells onto 1 mL of 20% sucrose in PBS followed by centrifugation at 20,000xg for 90 minutes at 4°C. Virus-containing pellets were resuspended in 40 μl 1X Laemmeli buffer and 10 μl was used for western blot analysis. Whole-cell extracts were also prepared at the same time to analyze intracellular Gag maturation.

## Supporting information

S1 Table Primer sequences. This table contains a list of primers used in this study.
(PDF)

S1 Fig CypA/RanCyp alignment and numbering reference. An alignment of human cyclophilin A (CypA) and human RanCyp. The full-length primary protein sequence of CypA is shown along with the RanCyp sequence that was used in this study for both functional and evolutionary analyses. Asterisks (*) represent sites that are conserved between CypA and RanCyp. Secondary structure motifs (PDB: 4I9Y, α- alpha helix, β- beta strand) are denoted above the alignment.
(PDF)

S2 Fig Alignment of lentiviral capsids used to generate cyclophilin-binding loop mutants. A) Sequences for the N-terminal domain of lentiviral capsids were collected from NCBI and aligned using ClustalX. Secondary structure motifs are indicated above the alignment along with numbering relative to HIV-1 NL4-3 strain. Asterisks (*) indicate conserved amino acid positions. The cyclophilin-binding loop is indicated with a gray box. B) Crystal structure of the N-terminal domain of HIV-1 capsid (pdb: 1AK4) with secondary structure motifs labeled. The cyclophilin-binding loop is colored red.
(PDF)

S3 Fig Alignment of cyclophilin A (CypA) from hominoids and old world monkeys. Amino acid alignment of cyclophilin A from primate species that encompass approximately 32 million years (Mya) of divergence time [51]. A cladogram representing the relationship between species is shown next to the alignment.
(PDF)

S4 Fig Sources (cell lines) of RanCyp and CypA sequences analyzed in this study. A) Sequences were either obtained from GenBank, assembled from the UCSC genome browser, or PCR amplified and sequenced manually (see **S1 Table** for primers). B) Source of RNA and DNA for RanCyp and CypA sequences and clones generated in this study.
(PDF)
