# Correction: Species-specific vulnerability of RanBP2 shaped the evolution of SIV as it transmitted in African apes

**DOI:** 10.1371/journal.ppat.1006983

**Published:** 2018-04-03

**Authors:** Nicholas R. Meyerson, Cody J. Warren, Daniel A. S. A. Vieira, Felipe Diaz-Griffero, Sara L. Sawyer

The fourth author's name is spelled incorrectly. The correct name is: Felipe Diaz-Griffero. The correct citation is: Meyerson NR, Warren CJ, Vieira DASA, Diaz-Griffero F, Sawyer SL (2018) Species-specific vulnerability of RanBP2 shaped the evolution of SIV as it transmitted in African apes. PLoS Pathog 14(3): e1006906. https://doi.org/10.1371/journal.ppat.1006906
