# Supplementary material for: Species-specific vulnerability of RanBP2 shaped the evolution of SIV as it transmitted in African apes
Source: PLoS Pathog. 2018 Mar 8;14(3):e1006906. doi: 10.1371/journal.ppat.1006906 (PMC5843284; doi:10.1371/journal.ppat.1006906)
Supplement: S1 Table — This table contains a list of primers used in this study. (PDF) [file ppat.1006906.s001.pdf]

Table S1. Primers used in this study

Primer Name	Primer Sequence (5' -> 3')	Details	
rm037	AAATGTGTATCATGCCAAAATCTAAACCCAAGC	RanBP2 amplicon (5684 bps) containing RanCyp domain	**Amplify and sequence *RanCyp* and *CypA***
NRM038	CTGCAATTTTACATTTGTAAATCGGAAACGTCC	Phusion in HF buffer + 3% DMSO - 58°C anneal - 1 min 40 s elongation	
NRM376	GAACCAATAGTGTCACTACCAGAGG	Sequencing primers for RanCyp domain	
NRM377	GGACTGCTGAGAACTTCAGAGC		
NRM445	TGGTATAAAAGGGGCGGGAGG	CypA amplicon (756 bps)	
NRM448	CATAAACTTAACTCTGCAATCCAGCTAGG	Phusion in HF buffer - 60°C anneal - 15s elongation	
NRM447	AACCCAAAGGGAACTGCAGCG	Sequencing primer for CypA	
NRM523	GGTGCTGGTGCTGGTGCT ATGGCTTCCAGAATCCTGGTCAATA	Owl monkey TRIM-Cyp RBCC fragment containing N-term linker for HA addition and C-term	**Generate TRIM-RanCyp chimeras**
NRM524	CCACAGGATTGGTCTCCTTTGATAAGGCTGATGCTACAAGGTCC	overlap for human, chimp, gorilla, orangutan, red-capped mangabey, and titi monkey RanCyp	
NRM525	CCACAGGATTGGTCTCCTTTGTTAAGGCTGATGCTACAAGGTCC	Used with NRM523 to generate TRIM-Cyp fragment with C-term overlap for marmoset RanCyp	
NRM526	GGACCTTGTAGCATCAGCCTTATCAAAGGAGACCAATCCTGTGG	RanCyp domains containing N-term overlap with owl monkey TRIM-Cyp RBCC fragment	
NRM527	GGACCTTGTAGCATCAGCCTTAACAAAGGAGACCAATCCTGTGG	NRM526/NRM510 - human, chimp, gorilla, orangutan, red-capped mangabey fragments	
NRM510	TCATATCTGTCCACATTCTGTGATAG	NRM527/NRM510 - marmoset fragment	
NRM511	TCAAATCTGTCCACATTCTGTGATAG	NRM526/NRM511 - titi monkey fragment	
NRM531	TTAAAGTTGTCCACAGTCAGCAATGG	Used with NRM523 to generate full-length owl monkey TRIM-Cyp with N-term epitope linker	
NRM530	TCGCCACCATGGGTTACCCATACGACGTCCCAGACTACGCCGGTGCTGGTGCTGGTGCT	Adds N-terminal HA tag	
NRM1136	CATGATGGAACAGGCAGACAGTCCATTTATGGAGACAAAT	G75R point mutation in the human TRIM-RanCyp background	**Generate TRIM-RanCyp mutants**
NRM1137	CATAAATGGACTGTCTGCCTGTTCCATCATGTTTGGTGAT		
NRM1138	CATGATGGAACAGGCGGACAGTCCATTTATGGAGACAGAT	R75G point mutation in the gorilla TRIM-RanCyp background	
NRM1139	CATAAATGGACTGTCCGCCTGTTCCATCATGTTTGGTGAT		
NRM1140	CCATTTATGGAGACAGATTTGAAGATGAAAATTTTGATGT	K82R point mutation in the human TRIM-RanCyp background	
NRM1141	TTTTCATCTTCAAATCTGTCTCCATAAATGGACTGTCCGC		
NRM1142	CCATTTATGGAGACAAATTTGAAGATGAAAATTTTGATGT	R82K point mutation in the gorilla TRIM-RanCyp background	
NRM1143	TTTTCATCTTCAAATTTGTCTCCATAAATGGACTGTCTGC		
NRM557	CTATCCATGGCCAATGAAGGCCAGAATACCA	Q103E point mutation in the human or chimp TRIM-RanCyp background	
NRM558	TGGTATTCTGGCCTTCATTGGCCATGGATAG		
NRM559	CTATCCATGGCCAATCAAGGCCAGAATACCA	E103Q point mutation in the gorilla TRIM-RanCyp background	
NRM560	TGGTATTCTGGCCTTGATTGGCCATGGATAG		
NRM1144	AATAATTCTCAATTTTTTATAACACTGAAGAAAGCAGAAC	V113F point mutation in the human TRIM-RanCyp background	
NRM1145	TCTTCAGTGTTATAAAAAATTGAGAATTATTGGTATTCTG		
NRM1146	AATAATTCTCAATTTGTTATAACACTGAAGAAAGCAGAAC	F113V point mutation in the chimp or gorilla TRIM-RanCyp background	
NRM1147	TCTTCAGTGTTATAACAAATTGAGAATTATTGGTATTCTG		
NRM1148	TCATTTGGTTCTCCCGAAGGGTCTGTTTGTCGAAGAATAA	K149E point mutation in the human TRIM-RanCyp background	
NRM1149	GACAAACAGACCCTTCGGGAGAACCAAATGATTCAATCTT		
NRM1150	TCATTTGGTTCTCCCAAAGGGTCTGTTTGTCGAAGAATAA	E149K point mutation in the gorilla TRIM-RanCyp background	
NRM1151	GACAAACAGACCCTTTGGGAGAACCAAATGATTCAATCTT		
NRM561	GGGATAGAGTGCATCCAGCGGTTGTAGGGCCTCTTCCAGTAGGCCAGATGAGAGAACC	Swaps SIVgor CP684 cyclophilin-binding loop into pMDLG backbone	**Generate capsid - cyclophilin binding loop chimeras**
NRM562	GGTTCTCTCATCTGGCCTACTGGAAGAGGCCCTACAACCGCTGGATGCACTCTATCCC		
NRM563	GGGATAGAGTGCATCCACAGCCTGTAGGGCCTCTTCCACCAGGCCAGATGAGAGAACC	Swaps SIVgor BP664 cyclophilin-binding loop into pMDLG backbone	
NRM564	GGTTCTCTCATCTGGCCTGGTGGAAGAGGCCCTACAGGCTGTGGATGCACTCTATCCC		
NRM609	AGAGTGCATCCAACGCATGCAGGGCCTCTTGTACCAGGCCAGATGAGAGAACC	Swaps SIVcpz CAM155 cyclophilin-binding loop into pMDLG backbone	
NRM610	ATCTGGCCTGGTACAAGAGGCCCTGCATGCGTTGGATGCACTCTATCCCATTCTGC		
NRM611	CATGCAGGGCCTGTTGCACCAGGCCAGATGAGAG	Swaps SIVcpz CAM3 cyclophilin-binding loop into pMDLG backbone	
NRM612	GGCCTGGTGCAACAGGCCCTGCATGCACTGGATG		
NRM613	AGAGTGCATCCAATCCATATGGGGCCTATTGCACCAGGCCAG	Swaps SIVcpz LB7 cyclophilin-binding loop into pMDLG backbone	
NRM614	TGCAATAGGCCCCATATGGATTGGATGCACTCTATCCCATTC		
NRM615	GAGTGCATCCAGCGCATGCAGGGCCTCAACAAGCAGGCCAGATGAGAGAACC	Swaps SIVcpz TAN cyclophilin-binding loop into pMDLG backbone	
NRM616	TCATCTGGCCTGCTTGTTGAGGCCCTGCATGCGCTGGATGCACTCTATCCC		
NRM669	AGAGTGCATCCAACGCATGCAGGGCCTGTACAAGCAGGCCAGATGAGAG	Swaps SIVcpz ANT cyclophilin-binding loop into pMDLG backbone containing SIVcpz TAN loop	
NRM670	TGGCCTGCTTGTACAGGCCCTGCATGCGTTGGATGCACTCTATCCCATTC		
NRM671	AGAGTGCATCCCAACCCTGGGCCTCTTCCAGCAGGCCAGATGAGAGAACCAAG	Swaps SIVrcm NG cyclophilin-binding loop into pMDLG backbone containing SIVgor BQ664 loop	
NRM672	TCATCTGGCCTGCTGGAAGAGGCCCAGGGTTGGATGCACTCTATCCCATTC		
NRM673	AGAGTGCATCCCAACCTTGGGCCTCTTCCAGCAGGCCAG	Swaps SIVrcm CAM cyclophilin-binding loop into pMDLG backbone containing SIVrcm NG loop	
NRM674	TGCTGGAAGAGGCCCAAGGTTGGGATGCACTCTATCCC		
NRM675	GAGTGCATCCCGTCCCTGGGCCTATTCCAGCAGGCCAGATGAG	Swaps SIVrcm GAB cyclophilin-binding loop into pMDLG backbone containing SIVrcm NG loop	
NRM676	CTGGCCTGCTGGAATAGGCCCAGGGACGGGATGCACTCTATCC		
NRM677	AGAGTGCATCCACAGCCTGCACCTCAACAAGGCCAGATGAGAGAACCAAG	Swaps SIVmac cyclophilin-binding loop into pMDLG backbone containing SIVcpz TAN loop	
NRM678	TCTCATCTGGCCTTGTTGAGGTGCAGGCTGTGGATGCACTCTATCCCATTC		
