# Supplementary figures and images for: Species-specific vulnerability of RanBP2 shaped the evolution of SIV as it transmitted in African apes
Source: PLoS Pathog. 2018 Mar 8;14(3):e1006906. doi: 10.1371/journal.ppat.1006906 (PMC5843284; doi:10.1371/journal.ppat.1006906)

Figure S1

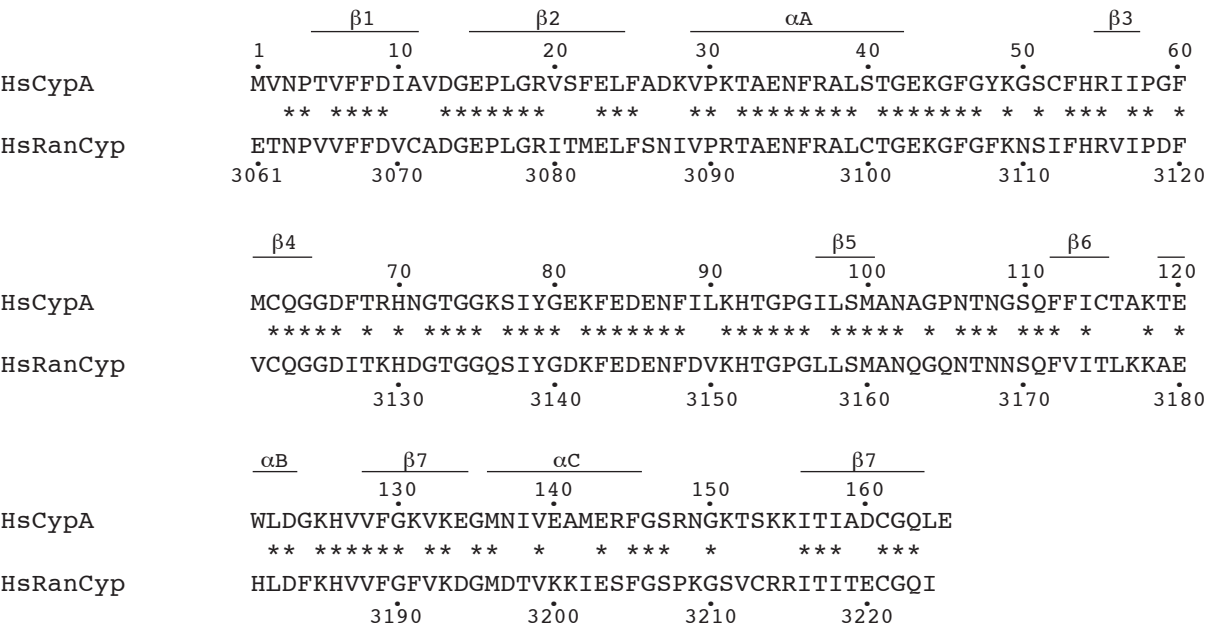

Supplement: S1 Fig — An alignment of human cyclophilin A (CypA) and human RanCyp. The full-length primary protein sequence of CypA is shown along with the RanCyp sequence that was used in this study for both functional and evolutionary analyses. Asterisks (*) represent sites that are conserved between CypA and RanCyp. Secondary structure motifs (PDB: 4I9Y, α- alpha helix, β- beta strand) are denoted above the alignment. (PDF) [file ppat.1006906.s002.pdf]

**Figure S3**

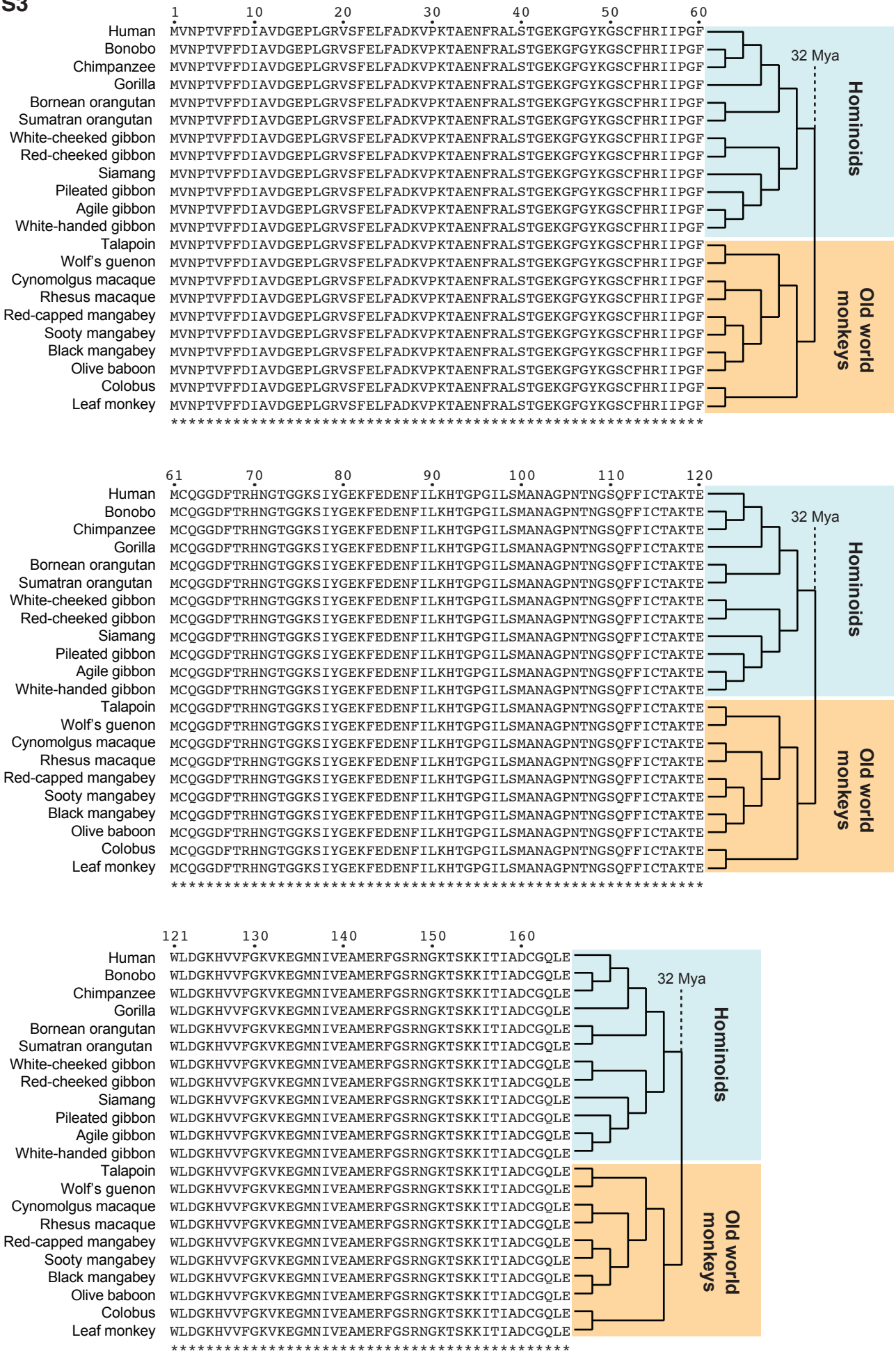

Supplement: S3 Fig — Amino acid alignment of cyclophilin A from primate species that encompass approximately 32 million years (Mya) of divergence time [51]. A cladogram representing the relationship between species is shown next to the alignment. (PDF) [file ppat.1006906.s004.pdf]
