# Supplementary material for: Species-specific vulnerability of RanBP2 shaped the evolution of SIV as it transmitted in African apes
Source: PLoS Pathog. 2018 Mar 8;14(3):e1006906. doi: 10.1371/journal.ppat.1006906 (PMC5843284; doi:10.1371/journal.ppat.1006906)

Figure S2

A

```
Accession                Beta Hairpin     α1            α2            α3
Numbers                           10         20         30         40         50         60
HQ179987  HIV_P          PIVQNAQGQMTHMPLSPRTLNAWVKAVEEKAFNPEIIPMFMALSEGAIPDDINTMLNAVG
FJ424871  SIVgorCP684    PVVTNAQGQMVHQPLSPRTLNAWVKAVEEKAFNPEIIPMFMALSEGSIPYDINTMLNAIG
FJ424868  SIVgorBQ664    PIIQNAQGQMVHQPLSPRTLNAWVKAVEEKAFSPEIIPMFMALSEGSLPYDINTMLNAIG
U26942    HIV_NL43       PIVQNLQGQMVHQAISPRTLNAWVKVVEEKAFSPEVIPMFSALSEGATPQDLNTMLNTVG
AF115393  SIVcpzCAM3     PVVQNAQGQLVHQPMSPRTLNAWVKVIEEKNFNPEVIPMFMALSEGATPQDVNTMLNAIG
AF447763  SIVcpzTAN1     PVITDAQGVARHQPISPRTLNAWVRVIEEKGFNPEVIPMFSALSEGATPYDLNSMLNAVG
AF349680  SIVrcmNG       PIVTINQ-QPEHQPISPRTLNAWVKVVEEKKFGAEVVPMFSALSEGCIPYDVNQMLNAIG
AF028608  SIVrcmGAB      PIITINQ-QPEHNPISPRTLNAWVKVVEEKKFSAEVAPMFSALSEGCIPYDINQMLNAIG
HM803689  SIVrcmCAM      PIITVNQ-QPEHQPISPRTLNAWVKVVEEKKFGAEVVPMFSALSEGCIPYDINQMLNAIG
AY588946  SIVmac239      PVQQIGG-NYVHLPLSPRTLNAWVKLIEEKKFGAEVVPGFQALSEGCTPYDINQMLNCVG
                         *          *   *********   *** *  *  * * *****  * * * ***  *

                                   α4                α5        α6
                                  70        80        90        100       110
          HIV_P          GHQGALQVLKEVINEEAAEWDRTHPVPVGPLPPGQLREPTGGDIAGTTSTKQEQITWMT-
          SIVgorCP684    GHQGALQVLKEVINEEAAEWDRQHPAVVGPLPVGQLREPSGGDIAGTTSTQQEQVAWMT-
          SIVgorBQ664    GHQGALQVLKEVINEEAAEWDRLHPQPVGPLPPGQIREPTGGDIAGTTSTQQEQISWTT-
          HIV_NL43       GHQAAMQMLKETINEEAAEWDRLHPVHAGPIAPGQMREPRGSDIAGTTSTLQEQIGWMT-
          SIVcpzCAM3     GHQGAMQVLKEVINEEAAEWDRTHPVHAGPVAPGQLREARGSDIAGTTSTLVEQIAWMT-
          SIVcpzTAN1     EHQAAMQMLKEVINEEAAEWDRAHPAHAGPQQAGMLREPTGADIAGTTSTLQEQVLWMTT
          SIVrcmNG       EHQGALQIVKEVINDEAADWDLRHPN-PGPLPAGQLREPTGSDIAGTTSNIAEQIAWTT-
          SIVrcmGAB      EHQGALQIVKEVINEEAADWDARHPV-PGPIPAGQLREPTGSDIAGTTSSIAEQIAWTT-
          SIVrcmCAM      EHQGALQIVKEVINEEAADWDARHPN-LGPLPAGQLREPTGSDIAGTTSSVAEQIAWST-
          SIVmac239      DHQAAMQIIRDIINEEAADWDLQHPQ-PAPQ-QGQLREPSGSDIAGTTSSVDEQIQWMY-
                          ** * *     ** *** **  **    *   *  **  * *******   **  *
                                                   Cyp Binding Loop

                                         α7
                         120        130       140
          HIV_P          -RNN-PVPVGDIYRKWIVLGLNKVVKMYCP
          SIVgorCP684    -RNNNPVPVGDIYRKWIVLGLNKVVKMYCP
          SIVgorBQ664    -RANGPIPVGDIYRKWIVLGLNKVVKMYCP
          HIV_NL43       -HNP-PIPVGEIYKRWIILGLNKIVRMYSP
          SIVcpzCAM3     -ANP-PVPVGEIYRRWVVLGLNKVVRMYCP
          SIVcpzTAN1     PQAQGGVPVGDIYKRWIILGLNKLVRMYSP
          SIVrcmNG       -RANNPIAVGNIYRNWIVLGLQKCVKMYNP
          SIVrcmGAB      -RANNPINVGNLYRNWIIVGLQKWVKMYNP
          SIVrcmCAM      -RANNPIIVGNIYRNWIILGLQKCVRMYNP
          SIVmac239      -RQQNPIPVGNIYRRWIQLGLQKCVRMYNP
                                 **  *  *   ** * * ** *
```

B

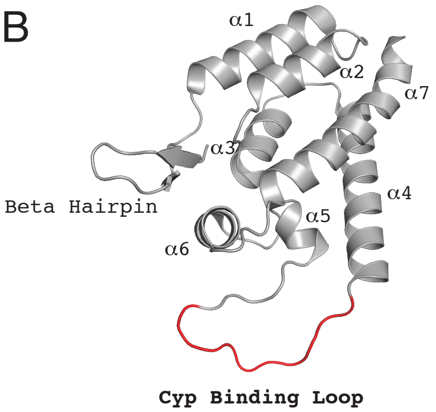

Supplement: S2 Fig — A) Sequences for the N-terminal domain of lentiviral capsids were collected from NCBI and aligned using ClustalX. Secondary structure motifs are indicated above the alignment along with numbering relative to HIV-1 NL4-3 strain. Asterisks (*) indicate conserved amino acid positions. The cyclophilin-binding loop is indicated with a gray box. B) Crystal structure of the N-terminal domain of HIV-1 capsid (pdb: 1AK4) with secondary structure motifs labeled. The cyclophilin-binding loop is colored red. (PDF) [file ppat.1006906.s003.pdf]
