# Supplementary material for: Species-specific vulnerability of RanBP2 shaped the evolution of SIV as it transmitted in African apes
Source: PLoS Pathog. 2018 Mar 8;14(3):e1006906. doi: 10.1371/journal.ppat.1006906 (PMC5843284; doi:10.1371/journal.ppat.1006906)
Supplement: S4 Fig — A) Sequences were either obtained from GenBank, assembled from the UCSC genome browser, or PCR amplified and sequenced manually (see S1 Table for primers). B) Source of RNA and DNA for RanCyp and CypA sequences and clones generated in this study. (PDF) [file ppat.1006906.s005.pdf]

Figure S4

A

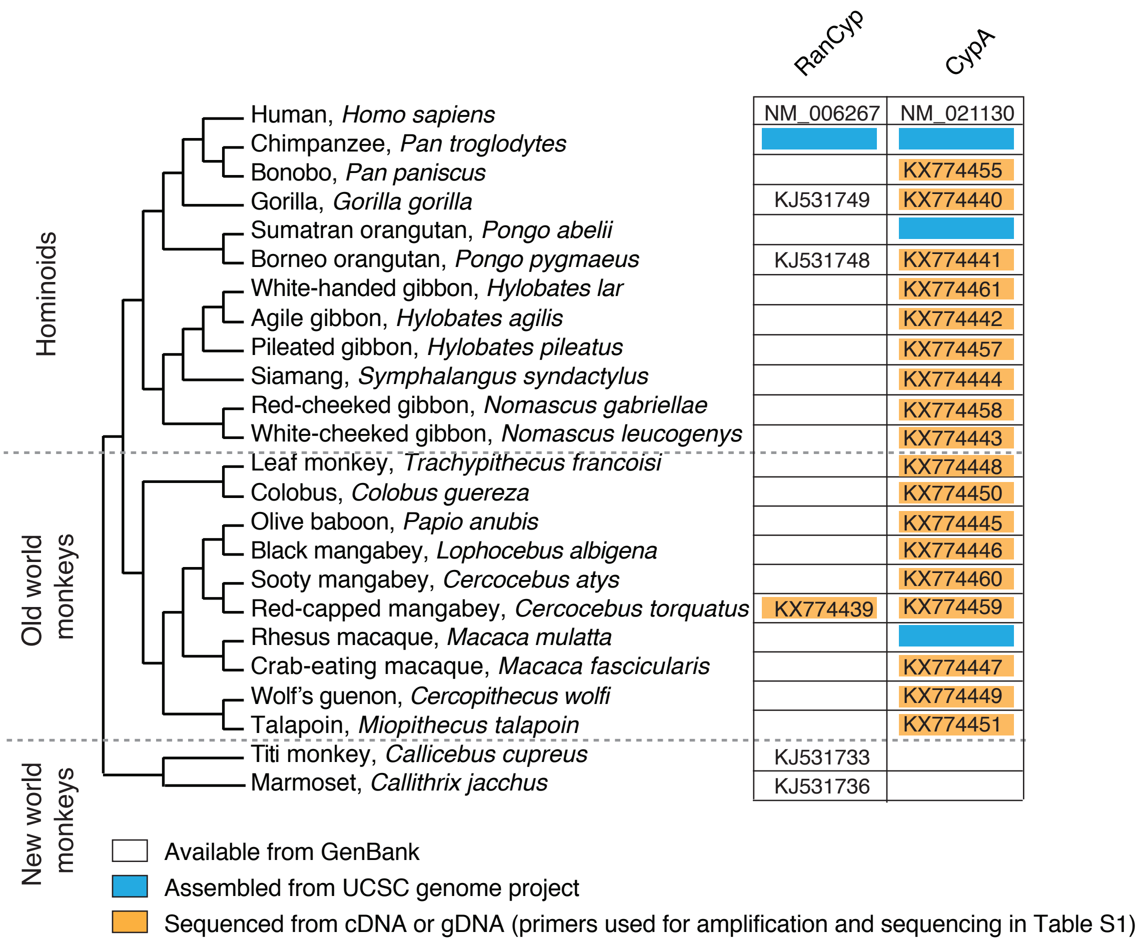

B

Source of DNA and RNA

Common Name	Species Name	Source	Unique ID	Cell type
Gorilla	*Gorilla gorilla*	Coriell	PR00280	Fibroblasts
Bonobo	*Pan paniscus*	Coriell	PR00748	B-Lymphocyte
Borneo orangutan	*Pongo pygmaeus*	Coriell	PR00650	B-Lymphocyte
Siamang	*Hylobates syndactylus*	Coriell	PR00722	Fibroblasts
White-cheeked gibbon	*Hylobates leucogenys*	Coriell	PR01037	Fibroblasts
Agile gibbon	*Hylobates agilis*	Coriell	PR00773	Fibroblasts
Red-capped mangabey	*Cercocebus torquatus*	Coriell	PR00485	Fibroblasts
Talapoin	*Miopithecus talapoin*	Coriell	PR00716	Fibroblasts
Colobus	*Colobus guereza*	Coriell	PR00980	Fibroblasts
Leaf monkey	*Trachypithecus francoisi*	Coriell	PR01099	Fibroblasts
Crab-eating macaque	*Macaca fascicularis*	NEPRC	Mf27-04	B-Lymphocyte
Olive baboon	*Papio anubis*	Coriell	PR00978	Fibroblasts
Black mangabey	*Lophocebus albigena*	Coriell	PR01215	Fibroblasts
Wolf's guenon	*Cercopithecus wolfi*	Coriell	PR01241	Fibroblasts
Marmoset	*Callithrix jacchus*	Coriell	GM07404	B-Lymphocyte
Titi monkey	*Callicebus cupreus*	Coriell	PR00793	Fibroblasts
White-handed gibbon	*Hylobates lar*	Coriell	PR01131	Fibroblasts
Red-cheeked gibbon	*Nomascus gabriellae*	Coriell	PR00381	Fibroblasts
Pileated gibbon	*Hylobates pileatus*	Coriell	PR00243	Fibroblasts
Sooty mangabey	*Cercocebus atys*	NEPRC	FJY	B-Lymphocyte

NEPRC = New England Primate Research Center
